# Patterning the Pore Orientation of Nanoporous Metal via Self‐Organization in Flow Cells

**DOI:** 10.1002/advs.202411695

**Published:** 2025-01-07

**Authors:** Congcheng Wang, Yang Li, Xiangwei Geng, Jiatao Mao, Qing Chen

**Affiliations:** ^1^ Department of Mechanical and Aerospace Engineering The Hong Kong University of Science and Technology Clear Water Bay Kowloon Hong Kong; ^2^ Department of Chemistry The Hong Kong University of Science and Technology Clear Water Bay Kowloon Hong Kong; ^3^ The Energy Institute The Hong Kong University of Science and Technology Clear Water Bay Kowloon Hong Kong

**Keywords:** dealloying, nanoporous metal, pore orientation, porous electrode, redox flow battery, self‐organization

## Abstract

Nanoporous metals, a class of free‐standing, high specific‐area materials, evolve from interface‐controlled self‐organization in a selective dissolution (e.g., dealloying). The process creates randomly oriented pores, in which slow mass transport has limited the functional applications of nanoporous metals. Here the control of the pore orientation is demonstrated with a dealloying analogy, reduction‐induced decomposition, achieved in flow cells. Via forced convection, the self‐organization is placed under the control of sufficiently rapid mass transport to suppress pore branching and align 100 nm‐wide ligaments and pores along the direction of reaction propagation, boosting the permeability by an order of magnitude while retaining the large surface area. The pore orientation can be further manipulated with a flow field for an orientation pattern akin to the expected fluid pattern, enabling a nanoporous silver electrode to deliver a peak power of 0.3 W cm^−2^ in a redox‐flow battery, outperforming commercial carbon electrodes.

## Graphical abstract

Percolation dissolution in a flow cell enables the control and even patterning of pore orientation of nanoporous metal as a highly permeable, nanostructured electrode.

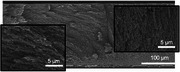

## 1 Introduction

Nanoporous (NP) metals have found broad significance as architected functional materials. They are fabricated via the selective dissolution of a homogeneous solid precursor, including electrochemical dealloying,^[^ ^1^ ^]^ liquid metal dealloying,^[^ ^2^ ^]^ and reduction‐induced decomposition (RID).^[^ ^3^ ^]^ These processes, though distinct in the chemistries, all leverage interface kinetics to organize metal atoms into networks of curvy ligaments a few to a few hundred nanometers wide and percolating pores of a similar length scale. The ligaments and pores are all contained in dimensions defined by the precursors (i.e., alloys in dealloying and compound crystals in RID), rendering bi‐continuous, free‐standing NP structures that suit many functional applications. For example, NP Au realized electrochemical actuation under stress up to 200 MPa,^[^ ^4^ ^]^ NP Pt delivered benchmarking electro‐catalytic activities toward oxygen reduction in fuel cells,^[^ ^5^ ^]^ and NP Zn achieved outstanding stability in rechargeable alkaline Zn batteries.^[^ ^6^ ^]^ Underlying the successes is our knowledge of how to precisely control structural characteristics. The microstructural length scales (the widths of ligaments and pores) depend on the surface diffusivity of the metal atoms, so thin surface oxides/salts can be exploited to freeze the length scales against coarsening for a high specific area.^[^ ^7^, ^8^ ^]^ The porosity (the volume fraction of pores) depends on the composition of the precursor. Although a high porosity remains challenging due to a loss of ligament connectivity, building a structural hierarchy has been an effective route leading to highly porous yet monolithic NP metals.^[^ ^3^, ^9^ ^]^

The structural tunability does not yet include the control of the orientation of the ligaments and pores. It is random for most NP metals, as evaluated roughly via electron microscopy or precisely via tomography and scattering techniques.^[^ ^10^, ^11^ ^]^ The random orientation roots in the interface‐controlled kinetics of morphology evolution during the fabrication. Recent work however shows that when the rate of dissolution exceeds the diffusional limits in the dissolution medium, the structure can indeed be aligned along the direction of dissolution. Such an observation was first made with liquid metal dealloying,^[^ ^12^ ^]^ in which the dissolution of Ta from a Ta‐rich Ti–Ta alloy creates oriented Ti lamella and filaments. Later we observed similarly that RID of AgCl, when occurring at a rapid rate, led to oriented NP Ag.^[^ ^13^ ^]^ Nonetheless, the observations have not enabled an orientation control for functional applications. For liquid metal dealloying, the fabrication temperature was too high to retain a small length scale, and the composition of the remaining component was too low to form a connected porous structure.^[^ ^2^ ^]^ For RID, the rapid rate was attained in a highly concentrated aqueous solution of NaBH_4_, where a large amount of H_2_ bubbles as a by‐product resulted in cracks. The diffusion‐limited reaction slows down when advancing deep into the precursor, so RID can only form oriented NP metal to a limited thickness.^[^ ^13^ ^]^ In neither case do we have a single nob to tune the orientation without significantly affecting other properties.

The importance of orientation control is tied to mass transports necessary for a pore‐filling fluid to access a metal surface deep in an NP monolith. In the literature, we have seen examples of NP metal catalysts with shallow utilized surface^[^ ^14^, ^15^ ^]^ and NP metal battery electrodes with low accessible capacities,^[^ ^16^ ^]^ likely associated with the random orientation of the nanopores that increases the tortuosity for mass transport. At the same time, we have seen very limited examples of NP metal as flow‐cell electrodes,^[^ ^15^, ^17^, ^18^ ^]^ because the tortuous nanopores are not expected to allow an electrolyte to flow without a large pressure loss.^[^ ^19^ ^]^ Moreover, electrolysis or energy storage in a scaled flow cell often uses a flow field to distribute an electrolyte via a pattern of flow.^[^ ^20^ ^]^ An electrode would provide the optimal path of transport if its pore orientation matched the pattern, a fabrication challenge not yet achieved even with complex procedures.^[^ ^21^ ^]^

We demonstrate here a precise control over the orientation of NP metal structures, achieved by RID in flow cells. NP Ag with ligaments and pores oriented along the propagation direction of RID can form by simply raising the flow rate without changing the compositions of either the dissolution medium or the precursor. We reveal the mechanism underlying the flow‐rate dependence of the orientation and characterize the hydraulic permeability of NP metals with different orientations. With a serpentine flow field in the flow cell, we can manipulate the propagation direction to generate a patterned orientation in NP Ag. As the pattern matches the flow path in the flow cell, the structure can be applied to the same cell as a highly permeable, high‐specific area electrode for a redox flow battery (RFB).

## 2 Flow‐Rate Controlled Structural Orientation

**Figure 1:**
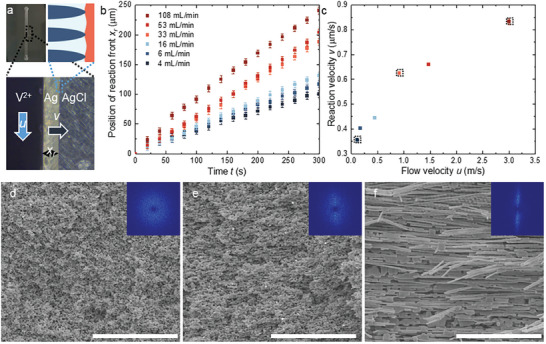
Flow‐rate controlled fabrication of NP Ag. a) The experimental setup (top left) and an optical microscopic image of an ongoing reaction (bottom). In the setup, the dissolution medium flows in the center channel (from the top to the bottom) shaped by a rubber gasket and flows by an AgCl sheet sandwiched between an acrylic plate and the gasket. The bottom image highlights the region around the reaction front, in which the light blue arrow corresponds to the direction of the solution flow and the dark blue one corresponds to the direction of reaction propagation. The cartoon (top right) illustrates the mechanism of structural evolution as proposed previously,^[^ ^13^ ^]^ with Ag in blue, AgCl in red, and the dissolution medium in light blue. b) The position of the reaction front (*x_r_*), i.e., the boundary between Ag and AgCl, captured at different durations of reaction time (*t*) at various flow rates. The position of the initial, unreacted edge of AgCl is set as *x_r_* = 0 (as shown in (a)). c) The reaction velocity (*v*) calculated from linear fittings to the results in (b). The flow velocity (*u*) is calculated from the flow rate, with the same color code as (b). d–f) The morphologies of NP Ag attained at *u* = 0.1, 0.9, and 3.0 µm s^−1^, respectively (the points in dashed boxes in (c). The scale bars are all 10 µm. The insets are 2D spectral density plots by Fourier transforming the images, each 80 µm^−1^ by 80 µm^−1^ with bright dots highlighting the characteristic length scales in the images.

We first demonstrate the connection between the flow velocity of a dissolution medium and the orientation of NP metal in RID‐based fabrication. The primary (half) reaction of concern is the reduction of AgCl, i.e., *AgCl* + *e* ^−^ → *Ag* + *Cl* ^−^ by a sufficiently strong reductant. In an aqueous solution without ions complexing the AgCl precursor, the reaction can be considered a selective dissolution of chloride ions, leaving behind Ag atoms that self‐organize at the interface between the reacting solid and the dissolution medium. Previously, we have shown that by raising the concentration of the reductant NaBH_4_, RID of AgCl can proceed under the control of diffusion in the dissolution medium (i.e., the aqueous solution of the reductant) to produce oriented NP Ag (referred to herein as Type‐I RID).^[^ ^13^ ^]^ However, the diffusion‐controlled reaction slows down with time. Below a rate threshold, the structure returns to typical NP Ag with a random orientation. So, we turn to forced convection. In a homemade flow cell (**Figure** **1**a; Figure S1, Supporting Information), a thin AgCl precursor is sandwiched between two acrylic plates, where space near the edge of the precursor is left open as a channel for the dissolution medium to flow by. By increasing the flow rate, we can accelerate the mass transport inside the evolving NP metal. Although we cannot estimate how effectively the flow can accelerate transport in the pores, we will show that it is sufficient to sustain a steady state of rapid reaction a few hundred microns deep.

Another notable change to our previous fabrication is the reductant. Instead of NaBH_4_, we use V^2+^ cations, prepared by electrolyzing a stock solution of VOSO_4_, a common practice for all‐vanadium RFBs.^[^ ^22^ ^]^ This change is to eliminate H_2_ bubbles generated during the oxidation of NaBH_4_, resulting in cracks in NP Ag and complicating the control of mass transport as we will discuss later. To dissolve a high concentration of V^2+^ for a high rate of reduction, we need to add 1 m H_2_SO_4_, a side effect of which is slight coarsening of NP Ag. The concentration of VSO_4_ is kept at 1 m in all cases, while the flow rate of the solution is used to control the rate of RID (referred to herein as Type‐II RID).

We can monitor the reaction propagation under an optical microscope due to the color difference between Ag and AgCl. Like in our previous work,^[^ ^13^ ^]^ the position of reaction front *x_r_* is estimated from images captured at a fixed time interval (20 s) and plotted against reaction time *t* in Figure 1b. At all six different flow velocities (*u*), *x_r_* displays a linear dependence on *t*, so the velocity of reaction front propagation *v* is derived as a constant in all the cases. The velocity (Figure 1c) increases from ≈0.35 µm s^−1^ at *u* = ≈0.1 m s^−1^ to above 0.8 µm s^−1^ at *u* = ≈3.0 m s^−1^, confirming the effectiveness of the flow for accelerating the reaction. They are however much lower than those measured in Type‐I RID.^[^ ^13^ ^]^ This difference will be discussed later along with the different structural outcomes.

The pore orientation of the NP structure indeed changes with the flow velocity. For *u* = 0.1 m s^−1^, the Ag ligaments have no preferred orientation in the scanning electron microscopic (SEM) image in Figure 1d. The mean width of the ligaments is ≈130 nm, larger than those prepared in NaBH_4_ solutions due to the different surface diffusivity of Ag at different pH values.^[^ ^23^ ^]^ The pores, complementing the ligaments in the material, should adopt a similarly random orientation, though difficult to visualize in SEM. At *u* = 0.9 m s^−1^, we observe a subtle orientation of the ligaments (Figure 1e). It can be better characterized with spectral density plots (the insets of Figure 1d–f), attained by Fourier transform of the binarized SEM images.^[^ ^24^ ^]^ Characteristic length scales show up as high‐intensity regions in the plots. For the NP Ag in Figure 1d, a ring in the spectral density shows the isotropy and thus the lack of orientation. For that in Figure 1e, the high‐intensity region is elliptical, as the ligaments elongate along the horizontal direction of the SEM image, that is, the direction of the reaction propagation. At the highest *u* of 3 m s^−1^, the ligaments are rather well‐aligned straight filaments (Figure 1f), whose length is not resolved in the spectral density plot as it is close to or longer than the SEM image. The filaments are much wider than the ligaments in Figure 1d,e, but if inspected closely, they are bundles of ligaments. The bundling is very likely due to the collapse of local porosity as the oriented ligaments are less connected, but the overall porosity is well retained as shown by the retention of the sample dimension.

The transition from the randomly oriented structure to the oriented one with increasing *v* is different from that in Type I.^[^ ^13^ ^]^ First, the threshold of *v* for the transition is nearly an order of magnitude lower (≈10 µm s^−1^ for Type I vs 0.5–0.9 µm s^−1^ for Type II). Second, the transition is gradual in Type II but sharp in Type I. The differences call for more scrutiny of the two types of RID. We first put the reaction velocities into the perspective of common electrochemical reactions. They can be converted to values of current density (*i*) via the Faraday's law,

(1) $$i=\frac{nFv}{V_{m}}\;$$

in which *n* is the number of electrons transferred (equal to one here), *F* the Faraday's constant, and *V_m_* the molar volume of AgCl. The highest value of *v* (≈0.5 µm s^−1^) in Type II converts to *i* of 0.2 A cm^−2^, typical for a limiting current density of a rapid electrochemical reaction (e.g., metal deposition) in a hydrodynamic method (e.g., rotating‐disk electrodes). The highest value of *v* (≈20 µm s^−1^) in Type I^[^ ^13^ ^]^ converts to *i* of 8 A cm^−2^, close to the limiting current of a gas evolution reaction (hydrogen evolution from water) where vigorous bubble formation forces convection. Therefore, we believe that both types rely on forced convection to speed up the reaction toward a condition for forming oriented NP metal, although the convection was unintentional in Type I. However, we do not consider the source of convection as the reason for the difference *v* thresholds for the orientation change.

Instead, the threshold difference more likely roots in the different length scales in the evolving porous structures. As we postulated previously^[^ ^13^ ^]^ and reiterate here, when the reaction is under the control of mass transport, pores are less prone to branching that would render long, tortuous paths and in turn slow down the growth of the pores. Oriented pores normal to the reaction front are thus favored. In this scenario, we compare two length scales to understand the velocity threshold; they are the diffusional layer thickness (*δ*) at the precursor/solution interface and the length scale at the reaction front (*λ*), the latter of which describes the distance between the branched pore tip and the adjacent ligament root. Here,

(2) $$\delta =DV_{m}\Delta C/v$$

where *D* is the diffusivity of reactant/product in the dissolution medium and Δ*C* the concentration gradient across the diffusional layer. Only when *δ* approaches *λ*, the diffusion‐controlled reaction rates between the pore tip and the ligament root become substantially different. As *δ* is inversely proportional to *v* and the other parameters in Equation (2) are similar or the same for Type‐I and ‐II RID's, we deduce that *λ* is much larger in Type II. As for what determines *λ*, we consider surface diffusivity the most important factor. *λ* here corresponds to the length scale at the moment of branching, which is different from the initial length scale of NP metal formation dictated by the precursor. *λ* is thus affected by how fast the structure coarsens. Therefore, the faster coarsening in the acid solution in Type‐II RID leads to larger *λ* and facilitates the formation of oriented ligaments and pores at larger *δ* and thus lower *v*. Note that the conclusion here seems to contradict our previous observation that Type‐I RID in a strongly alkaline solution,^[^ ^13^ ^]^ which suppresses coarsening but lowers the *v* threshold for the orientation change. We can only speculate that the high alkalinity may affect more than the surface diffusion, as the precursor AgCl is chemically unstable at high pH.

The gradual transition from the random to oriented structures with the flow rate *u* can be due to the competition between ligament growth and pinchoff. At steady‐state *v*, a ligament would continue to grow straight, unless surface diffusion pinched it off at the reaction front to reduce the overall energy contributed by the incoherent interface between Ag and AgCl. As *v* increases with *u*, the straight ligament can grow longer before being pinched off, the frequency of which is determined by the rate of surface diffusion.^[^ ^25^ ^]^ We did not see the dependence of the orientation on *v* in Type I, likely because the less frequent pinchoff, inferred from the narrow ligaments, cannot catch up with the rapid reaction.

## 3 Orientation‐Dependent Permeability

Before exploring the functionality, let us examine how the orientation can impact transport properties. As we intend the oriented NP structure to serve as an electrode in a flow cell, one transport property of interest is the hydraulic permeability (*K*), as defined in the Darcy's law

(3) $$K=\frac{\mu QL}{A\Delta P}$$

where *Q* is the volumetric flow rate, *μ* is the viscosity of the fluid, *A* is the geometric area of the electrode in the form of a thin sheet normal to the flux of the flow, Δ*P* is the differential pressure, and *L* is the electrode thickness. The orientation affects *K* through the tortuosity (*τ*) of the pores, that is, the pathlength of fluid with respective to the thickness. Two other key structural characteristics are the porosity (*ε*) and the pore width (*d*). *K* is intuitively proportional to *ε* (i.e., only the areal fraction of pores is considered permeable), and proportional to the square of a characteristic length scale, taken here as *d* as in the Hagen−Poiseuille law. Therefore, a correlation exists in the form of

(4) $$K\propto \frac{\varepsilon d^{2}}{\tau^{2}}$$

The reason τ is raised to a power of two was explained by Epstein.^[^ ^26^ ^]^ Equation (4) is also consistent with a rigorous derivation by Avellaneda and Torquato.^[^ ^27^ ^]^ A general tradeoff thus exists between small *d* and large *K*; the former is for providing a high specific area and the latter is for accessing the electrode surface at a reasonable flow rate.

**Figure 2:**
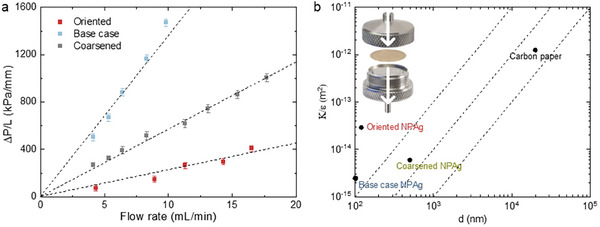
Structural‐dependence permeability. a) Thickness‐normalized pressure drops (Δ*P/L*) measured for three different NP Ag structures at different flow rates. b) Hydraulic permeability (*K*), normalized by porosity *ε* for both NP Ag's and carbon paper, plotted against the characteristic pore width *d*. The inset is an exploded view of the measurement device with the white arrows showing the direction of water flow.

This tradeoff can be partly addressed by lowering *τ* with oriented NP Ag, as shown by permeability measurements (**Figure** **2**). The measurements employ a Swagelok‐type flow‐through cell (the inset of Figure 2b), in which a disk of NP Ag is placed between a stainless‐steel gauze and a rubber O‐ring that sets the value of *A*. As water is flown through the cell at different rates, the pressure difference between the inlet and the outlet is recorded as Δ*P*. The measurements are made for three types of NP Ag, as shown in Figure 2a. The first is the oriented NP Ag whose ligaments and pores are aligned with the flow direction prepared by Type‐II RID (Figure 1f). The second is the NP Ag with a random orientation (that in Figure 1d), termed herein as the base case. The third is prepared by coarsening the base case at 200 °C, which increases the ligament and pore widths to ≈420 nm while retaining the random orientation (Figure S2a, Supporting Information). We caution that the three structures do not have the same value of *ε* and *L* (Table S1, Supporting Information), due to volume shrinkage during RID and coarsening. The base case has the highest *ε* of 61%, which is brought down by the coarsening to 58%. *ε* of the oriented structure is 55%, likely because the straight ligaments are less connected to uphold the structure locally as mentioned earlier. Despite having the lowest *ε*, the oriented structure displays the lowest Δ*P* among the three. At a moderately low flow rate of 10 mL min^−1^, the pressure drops by as much as 1400 kPa mm^−1^ through the base case, and it is reduced to 580 and 220 kPa mm^−1^ for the coarsened and oriented structures, respectively. Δ*P* appears to rise linearly with *Q* given the low Reynold's number for a transition toward a non‐Darcy behavior due to both the relative low flow rate and the small pores. We thus fit the results to the Darcy's law for *K* values.

In Figure 2b, we plot the values of permeability *K* against the pore width *d* for the three NP structures, which are compared with carbon paper, one of the most common commercial porous electrodes (Figure S2b, Supporting Information). In the plot, *K* is normalized by ε, so we can focus on the effects of *τ* and *d*. A set of dashed lines is also shown for the relationship of *K*∝*d* ^2^ (Equation (4)). It is not a surprise that the points do not aggregate on a single dashed line given the distinct morphologies; even the coarsened NP Ag is different from the base case as the nanocrystalline structure necks at grain boundaries during the coarsening. Nonetheless, the oriented NP Ag stands out, close to the top left region desired for the high *K* and small *d*. *K/ε* of the oriented NP Ag is ≈12 times higher than that of the base case, with which we can derive via Equation (4) that the ratio of *τ* between the two is ≈3.5. As the minimal possible *τ* value is 1, it suggests *τ* of the base case NP Ag to be >3.5, a high value that underscores the difficulty of using it in a flow cell. This value is much larger than what we measured for ionic diffusion in the same structure (1.8 for the base case and 1.3 for the oriented structure),^[^ ^28^ ^]^ which we speculate to originate from the different length scales associated with the transport processes (e.g., the mean free path of ions versus the diffusional layer thickness). For carbon paper, *τ* is previously estimated to be ≈1.2–1.3,^[^ ^29^ ^]^ so its high *K/ε* over the oriented structure benefits mainly from the larger pore size.

## 4 Patterned Orientation

**Figure 3:**
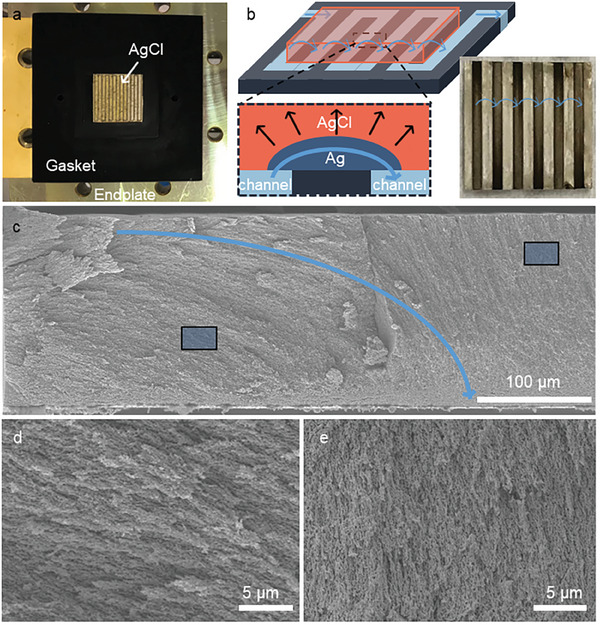
Fabrication of NP Ag with a patterned orientation. a) The setup, with AgCl placed on the serpentine flow field (visible through the translucent AgCl) and surrounded by a rubber gasket. Another endplate (not shown) will be pressed on top to seal the setup during the fabrication. b) A schematic of the fabrication process. The dissolution medium (light blue) is flown through the channel of a flow field (gray), on top of which lies AgCl (red). We magnify a fraction of the reacting cross‐section at the bottom, where the light blue arrows show the direction of solution flow and the dark blue arrows suggest the direction of reaction propagation. The bottom right corner is a photo of an as‐fabricated, 5 cm^2^ large NP Ag. c–e) The cross‐sectional morphologies. (d) and (e) are magnified views of the highlighted regions in (c). The orientation in (c) changes roughly along the blue arrow, which is the expected flow path.

While the oriented NP Ag fabricated so far can readily serve in flow cells with similarly oriented, straight flow paths (e.g., a Swagelok cell^[^ ^17^ ^]^), we leverage the control over the orientation for a bigger challenge associated with complex flow paths. The flow path we choose here arises from a serpentine flow field (**Figure** **3**a; Figure S3, Supporting Information), one of the most common in RFBs and electrolyzers.^[^ ^20^ ^]^ Fluid is flown through a serpentine channel engraved in a plate and spontaneously forced into a porous electrode by the pressure difference between adjacent parallel segments of the channel, illustrated in the cartoon in Figure 3b. The segments can be considered as the respective inlet and outlet for the portion of porous electrode atop them, setting a flow pattern as shown by the blue arrows. Pores in neither commercial nor advanced electrodes (e.g., those fabricated by electrospinning^[^ ^21^ ^]^) can yet match such a pattern. Oriented NP Ag has an edge. Its orientation is perpendicular to the reaction front of RID, which is controlled by how we expose a precursor to a dissolution medium. Exposing one side of a cuboid, as we have done so far, leads the reaction to propagate toward a single direction, which gives us NP Ag of a single orientation. Exposing parts of the surface instead can create a patterned orientation. As illustrated with an example in Figure 3b, if we place a precursor on top of the field filled with a flowing dissolution medium, the ligaments and pores of the as‐evolved NP metal will first radiate from the channel segments and then follow roughly the streamlines of the flow between the segments. We expect the orientation pattern to match the flow pattern, as both the reaction front of diffusion‐controlled RID and the fluid flow seek the shortest distance between the source and sink of the respective transport process.

We implement this fabrication as follows. The precursor, a 200 µm‐thick, 2.25 cm‐wide square AgCl sheet, is pressed against a graphite flow plate with a serpentine flow field and sealed with rubber gaskets to limit the initial contact with the dissolution medium to only the area above the channel (Figure 3a). 1 m V^2+^ is then flown through the assembled flow cell at 60 mL min^−1^ to render RID. The product displays a pattern of distinct colors (Figure 3b) in the areas above the channel and the graphite landing (i.e., the solid part of the flow field between the channel segments), respectively, underlaid by the patterned orientation. The surface of the darker gray regions comprises ligaments and pores perpendicular to the plane of view formed on top of the channels, whose higher roughness scatters light more heavily, whereas the lighter gray regions comprise those in the plane of view with lower roughness. The orientation distribution is revealed at the cross‐section under SEM (Figure 3c–e). The ligaments formed above the graphite curve toward the nearby channel (Figure 3d), whereas those above the channel are aligned vertically, perpendicular to the flow plate (Figure 3e), in excellent agreement with our postulation. The width and the aspect ratio of the ligaments are similar to that in Figure 1f, independent of the orientation, showing the consistency of the fabrication method underpinned by the controlling evolution kinetics.

## 5 RFB Electrode

**Figure 4:**
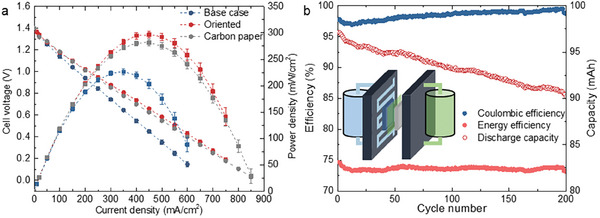
The application of NP Ag in a RFB. a) Polarization curves during discharging fully charged RFBs with three different porous electrodes in the negative sides, respectively. Power density, the product of the voltage and the current density is plotted against the right axis. b) Coulombic and energy efficiencies (dots) and discharging capacity (circles) versus cycle number achieved by the oriented NP Ag in the RFB. The inset illustrates the build of the battery, comprising a bottle of negative electrolyte, a negative flow plate, the porous negative electrode of interest, an ion‐selective membrane (Nafion), carbon paper as the positive electrode, a positive flow plate, and a bottle of positive electrolyte in sequence from left to right.

We apply this patterned NP Ag in a redox flow battery with the same serpentine flow field, the build of which is illustrated in **Figure** **4**. Given the similarity between the flow path and the orientation pattern, we expect the RFB to benefit from the low permeability as measured for the oriented NP Ag in Figure 2b. Though still less permeable than the commercial electrodes with large pores, its high specific area can boost the current output when it is limited by reaction kinetics. The RFB here comprises dihydroxyanthraquinone (DHAQ) in the negative electrolyte and ferrocyanide in the positive electrolyte, both with KOH as the supporting electrolyte.^[^ ^30^ ^]^ Only in the negative side do we employ NP Ag, which will be oxidized in the positive side. We choose this RFB chemistry for the following reasons. First, the reduction potential of DHAQ is slightly above hydrogen evolution, so we can avoid this side reaction on a good electrocatalyst like Ag. Second, the crossover of ferricyanide from the positive side, which may react with Ag in the negative side, is very slow through a typical ion‐selective membrane like Nafion. Third, the RFB chemistry uses all commodity chemicals, which facilitates comparisons with other work.

The oriented NP Ag can deliver a higher current and power than the carbon paper, as evaluated in polarization experiments. In the experiments, the RFB is first fully charged and then discharged at different currents to attain the discharging voltages. The steady‐state voltage is plotted against the current density in Figure 4a and Figure S4 (Supporting Information) for flow rates in the range of 16–120 mL min^−1^. In addition to the oriented NP Ag, we test the base‐case NP Ag and the carbon paper as controls, the latter of which has been widely used in similar RFBs. At a low flow rate (e.g., 16 mL min^−1^), both NP Ag electrodes display low mass‐transport limited current density that plateaus toward 400 mA cm^−2^ (Figure S4, Supporting Information), as the relatively low permeability drives the electrolyte to bypass the porous electrode in the serpentine flow field. As a result, the electrolyte cannot access the full surface area of the porous electrode, under‐utilizing the high specific area. A high flow rate (e.g., 120 mL min^−1^) boosts the currents for all, but the effect is the most significant with the oriented NP Ag (Figure 4a). Its current density and peak power density even exceed those of the carbon paper, despite the lower permeability. Although we cannot yet quantify the relationship between the current density and the earlier measured electrode characteristics (*K* or *d*), the results are promising. The comparison between the oriented and the base‐case NP Ag's shows the effectiveness of the orientation patterning, whereas the comparison between the oriented NP Ag and the carbon paper demonstrates the benefit of a high specific area. We note that the electric conductivity does not lead to the difference, even though the NP Ag electrodes are much more conductive (≈10^5^ S cm^−1^), because the typical resistance contribution from the less conductive carbon paper is ≈10 mΩ cm^2^,^[^ ^31^ ^]^ less than 1% of the overall polarization resistance.

The oriented NP Ag can serve in the RFB for more than 120 h, undergoing 200 stable galvanostatic cycles of charging and discharging (Figure 4b). The stable performance roots in the structural stability, which is confirmed by characterizing the NP Ag electrode harvested from the tested RFB (Figure S5, Supporting Information). We attribute the stability partly to how NP metal was formed; surface diffusion, the main mechanism of coarsening, has actively shaped NP metal into a steady‐state structure robust against further coarsening.^[^ ^32^ ^]^ Moreover, the RFB operates with an alkaline electrolyte, known to result in slower surface diffusion than an acidic electrolyte where NP Ag was prepared.^[^ ^23^ ^]^ When comparing the electrochemical impedance spectra before and after the cycling (Figure S7, Supporting Information), we can observe a slight increase in the high frequency value, which can be from the change in the membrane, as the electrode displays little change in the structure (Figure S5, Supporting Information). The energy efficiency is very close to the literature values attained with the carbon paper.^[^ ^30^ ^]^ The Coulombic efficiency starts low, but it climbs gradually above 99% after 100 cycles. The loss of the Coulombic efficiency is unlike due to the decomposition of DHAQ catalyzed by NP Ag, as the capacity decay is very similar to that with the carbon paper^[^ ^30^, ^33^ ^]^ and independent of the cycle number. We suspect hydrogen evolution to be the cause, which can be catalyzed by Ag more than carbon at a sufficiently high charging voltage.

## 6 Discussion

Though demonstrated in a RFB, the application of oriented NP metal can potentially be more significant in other flow devices. In our case, the electron transfer kinetics of DHAQ is already fast^[^ ^30^ ^]^ and its reduction potential is close to that of water, resulting in a limited benefit from a high‐specific area, catalytic electrode, possibly outweighed by the loss of Coulombic efficiency. In contrast, many sluggish reactions in flow electrolyzers can make better use of it. Among them are the electrochemical reduction of CO_2_^[^ ^34^ ^]^ and the hydrogenation of organic molecules,^[^ ^35^ ^]^ which have been shown to benefit greatly from nanostructured metal electrodes. Compared to those demonstrated in the literature, our oriented NP metal offers a unique combination of transport and surface properties to serve as an alternative to common metal foams and carbon electrodes.

There are many paths forward to optimize oriented NP metal to better meet the requirements of a flow cell. The two most intuitive paths are to enlarge the pores and increase the porosity. While we can routinely attain large pores in NP metals with coarsening,^[^ ^36^ ^]^ the method is not yet established for the oriented structure. Our preliminary test shows that coarsening does increase the length scale (Figure S6, Supporting Information) but at the expense of uniformity and porosity. The porosity of NP metal so far in this work is low compared to common flow‐cell electrodes, as its relative density (1–ε) is ultimately limited by percolation below which the structure falls apart.^[^ ^9^, ^32^ ^]^ For the case of AgCl RID, we can in principle raise the content of dissolving components by adopting an alloy precursor (e.g., Ag_0.75_Na_0.25_Cl),^[^ ^3^ ^]^ but the product, though still oriented NP Ag, is very fragile due to low ligament connectivity. A remedy is structural hierarchy,^[^ ^3^, ^9^ ^]^ which in fact may satisfy both the needs for large pores and high porosity, a promising approach that should be explored for oriented NP structures. Another optimization pertains to the composition of NP metal, which can be tuned through the precursor composition or a galvanic replacement reaction that deposits more noble metal on NP metal.

In short, through transport‐controlled self‐organization in flow cells, we demonstrate the fabrication of NP metal of a patterned orientation that can readily compete with commercial RFB electrodes. The material can find its use in many other flow devices that desire large catalytic metal surface after further structural optimization based on the insights into the structural evolution mechanism.

## 7 Experimental Section

### Sample Preparation

AgCl precursors were prepared by melting AgCl powder synthesized by mixing solutions of AgNO_3_ (Sigma–Aldrich) and NaCl (VWR). The melting process was carried out in a quartz crucible at 500 °C for 15 min in air. AgCl sheets were obtained by cutting and cold rolling the soft AgCl crystal. For reaction rate measurements, the AgCl sheet was cut into a rectangular shape and assembled into the flow cell. For permeability measurements, the AgCl sheet was cut into a round shape with a diameter of 12 mm by a steel puncher. An aqueous solution of 1 m VSO_4_ (prepared from VOSO_4_, Haizhongtian Fine Chemical Factory, Shenyang) and 1 m H_2_SO_4_ (Sigma–Aldrich) was pumped into the cell as the reducing agent and the NP Ag product after RID retained the shape. The Ag product was confirmed by X‐ray diffraction (Figure S8, Supporting Information). For the patterned orientation, a 5 cm^2^ AgCl sheet (300 µm thick) was assembled into zero‐gap flow battery hardware (Fuel Cell Tech., Albuquerque, USA). The same reducing solution was flown through a serpentine graphite flow plate, against which the AgCl sheet was pressed tightly. Carbon paper (SGL 39AA, Sigracet) was baked in air at 400 °C for 24 h before experiments. Copper foam (Suzhou Keshenghe Metals) was used as received.

### Microscopy Characterizations

Scanning Electron Microscopy (SEM) was conducted with a JEOL‐7100F Ultra‐high Resolution Scanning Electron Microscope. All SEM images were taken at the cross‐sections of nanoporous metals. All samples were thoroughly washed with deionized water and ethanol before characterization.

### Spectral Density Analysis

A SEM image was binarized and subtracted off the mean pixel value to attain an image of local fluctuations as implemented in ref. [24] This image then underwent fast Fourier transform assuming a periodic boundary condition to afford a spectral density plot.

### Reaction Rate Measurements

The AgCl sheet precursor was placed in an acrylic flow cell under an optical microscope (ZZW‐3000HU, Shenzhen Zhongzheng Instrument). The moving reaction front was recorded during the RID reaction. After recording the reaction, the same analysis method was applied as that in ref. [13] to attain the reaction front position.

### Fluid Permeability Measurements

A stainless‐steel flow cell was used to measure the fluid permeability. All the electrodes were in round shape with a diameter of 12 mm and the effective diameter measured in this cell was 10 mm. The Deionized water was pumped through the flow cell at rates controlled by a syringe pump and recorded the pressure drop of the flow cell with a pressure gauge.

### RFB Tests

The battery employed the same hardware as that in the fabrication of NP Ag of the patterned orientation. In the battery, the negative electrode was either the base‐case NP Ag, the oriented NP Ag, or three sheets of carbon paper (compressed to the same thickness as NP Ag, ≈300 µm). The positive electrode was always the carbon paper. The electrode area was 5 cm^2^ with the remaining flow plate area covered by rubber gaskets as in a typical zero‐gap configuration. The positive electrolyte was prepared by dissolving potassium ferrocyanide trihydrate in 1 m KOH (VWR) solution to afford a 0.4 m ferrocyanide and 2.6 m potassium electrolyte solution. The negative electrolyte was prepared by dissolving 2,6‐DHAQ (Sigma–Aldrich) in 2 m KOH solution (5 mL) resulting in a 0.5 m 2,6‐DHAQ and 1 m potassium electrolyte solution. Before the tests, the electrolytes were purged with ultra‐high purity argon for 1 h to ensure deaeration. To obtain the polarization curves, the cell was first charged to the desired SOC. Then the cell was discharged at constant currents for more than 30 s and recorded the steady‐state discharge voltages, during which the voltage variation was used to estimate the error.

## Conflict of Interest

The authors declare no conflict of interest.

## Supporting information



Supporting Information

## Data Availability

The data that support the findings of this study are available from the corresponding author upon reasonable request.
