# Supplementary material for: Patterning the Pore Orientation of Nanoporous Metal via Self‐Organization in Flow Cells
Source: Adv Sci (Weinh). 2025 Jan 7;12(8):2411695. doi: 10.1002/advs.202411695 (PMC11848635; doi:10.1002/advs.202411695)
Supplement: Supplementary file 1 — Supporting Information [file ADVS-12-2411695-s001.docx]

**Supporting Information**

**Patterning the pore orientation of nanoporous metal via self-organization in flow cells**

Congcheng Wang,^1^ Yang Li,^1^ Xiangwei Geng,^1^ Jiatao Mao,^2^ and Qing Chen^1,2,3*^

^1^Department of Mechanical and Aerospace Engineering, the Hong Kong University of Science and Technology, Clear Water Bay, Kowloon, Hong Kong.

^2^Department of Chemistry, the Hong Kong University of Science and Technology, Clear Water Bay, Kowloon, Hong Kong.

^3^The Energy Institute, the Hong Kong University of Science and Technology, Clear Water Bay, Kowloon, Hong Kong.

*Correspondence addressed to [chenqing@ust.hk](mailto:chenqing@ust.hk).


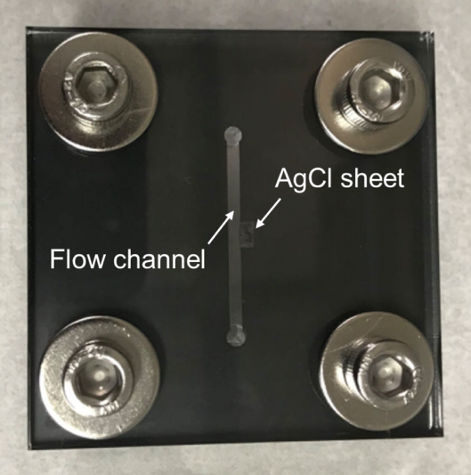


Figure S1. A photo of the flow cell used for Type-II RID.


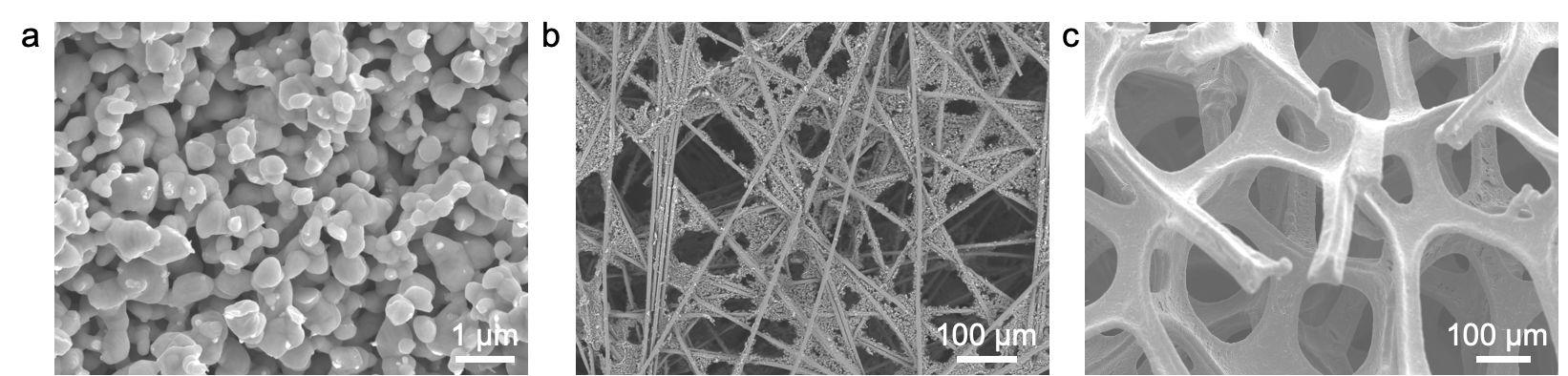


Figure S2. Porous structures used in the permeability measurement. (a) NP Ag attained by coarsening the base case at 200 ºC for 1 hour. (b) Carbon paper, baked in air at 400 ºC for 24 hours to ensure its wettability.

Table S1. Properties of all the porous structures undergoing the permeability measurement. *K/ε* for copper foam is 5.2×10^-10^ m^2^, which is not included in Fig. 2b.

|  | Pore width *d* (nm) | Porosity ε | Thickness *L* (µm) |
| --- | --- | --- | --- |
| Base case NP Ag | 100 | 0.61 | 53 |
| Coarsened NP Ag | 420 | 0.58 | 51 |
| Oriented NP Ag | 116 | 0.55 | 48 |
| Carbon paper | 2.0×10^4^ | 0.89 | 280 |
| Copper foam | 4.0×10^5^ | 0.95 | 1000 |


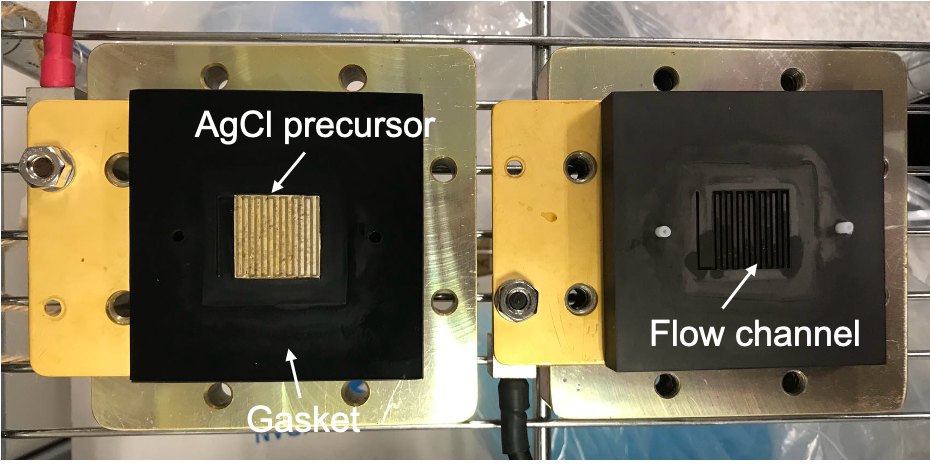


Figure S3. A photo of the serpentine flow plate. The inlet is connected to the top left tip of the channel from the back of the plate and the outlet to the bottom right tip.


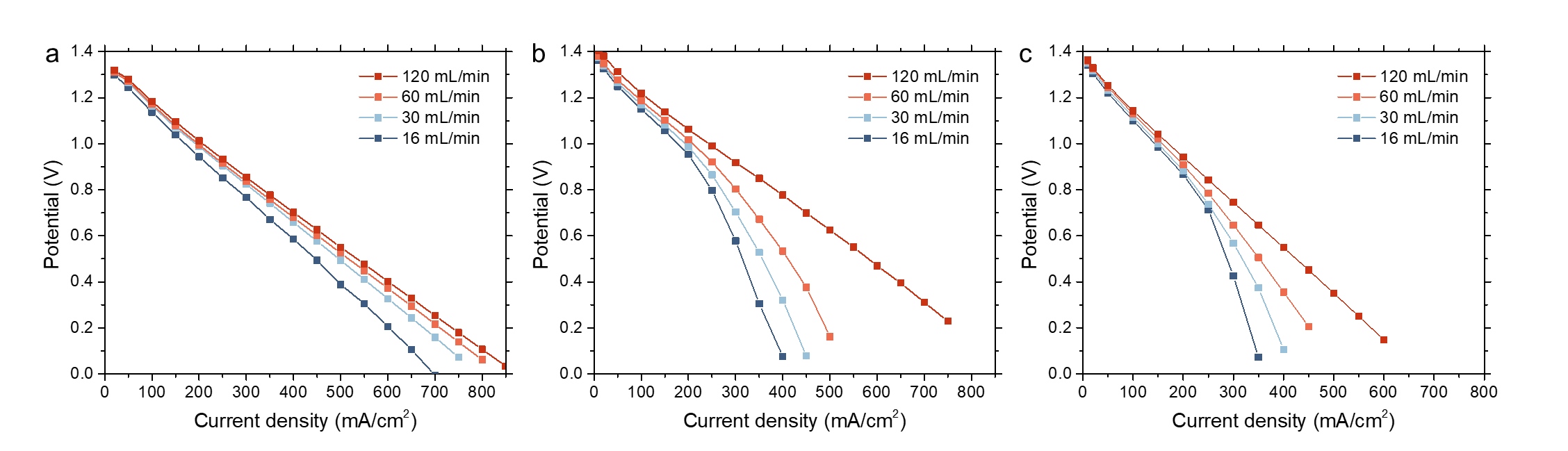


Figure S4. Polarization curves of (a) carbon paper, (b) oriented NP Ag, and (c) base-case NP Ag.


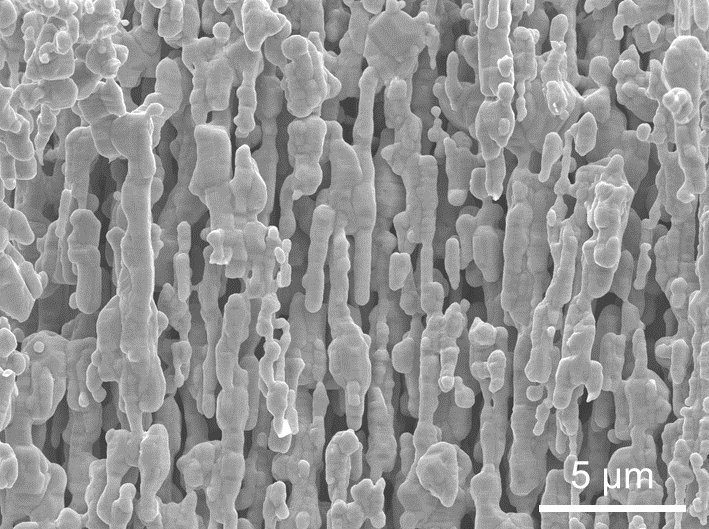


Figure S5. The morphology of oriented NP Ag after the cycling test shown in Fig. 4b.


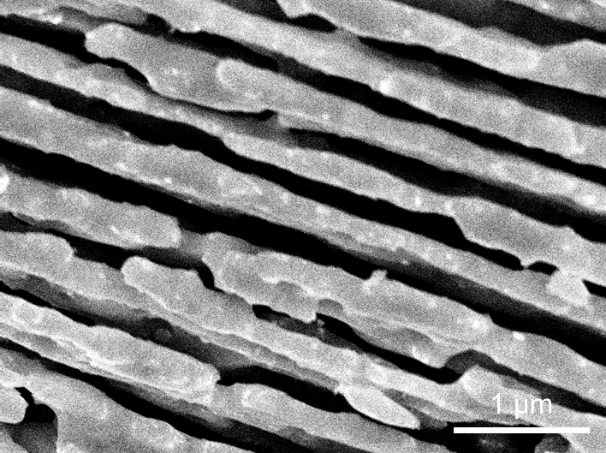


Figure S6. The morphology of oriented NP Ag after coarsening at 200 ºC for 1 hour.


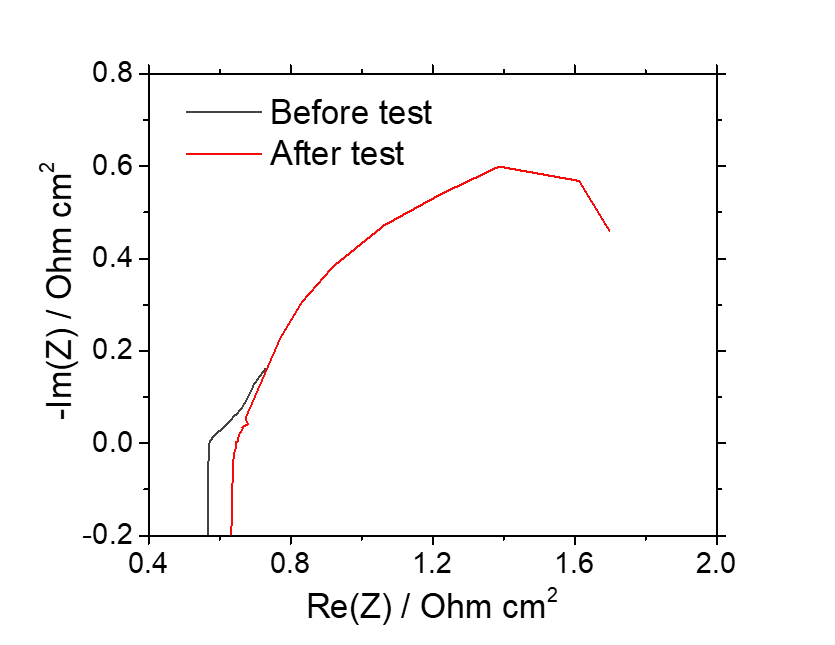


Figure S7. Electrochemical impedance spectroscopy (Nyquist plot) of the flow battery cell with NP Ag electrode before and after the cycling test.


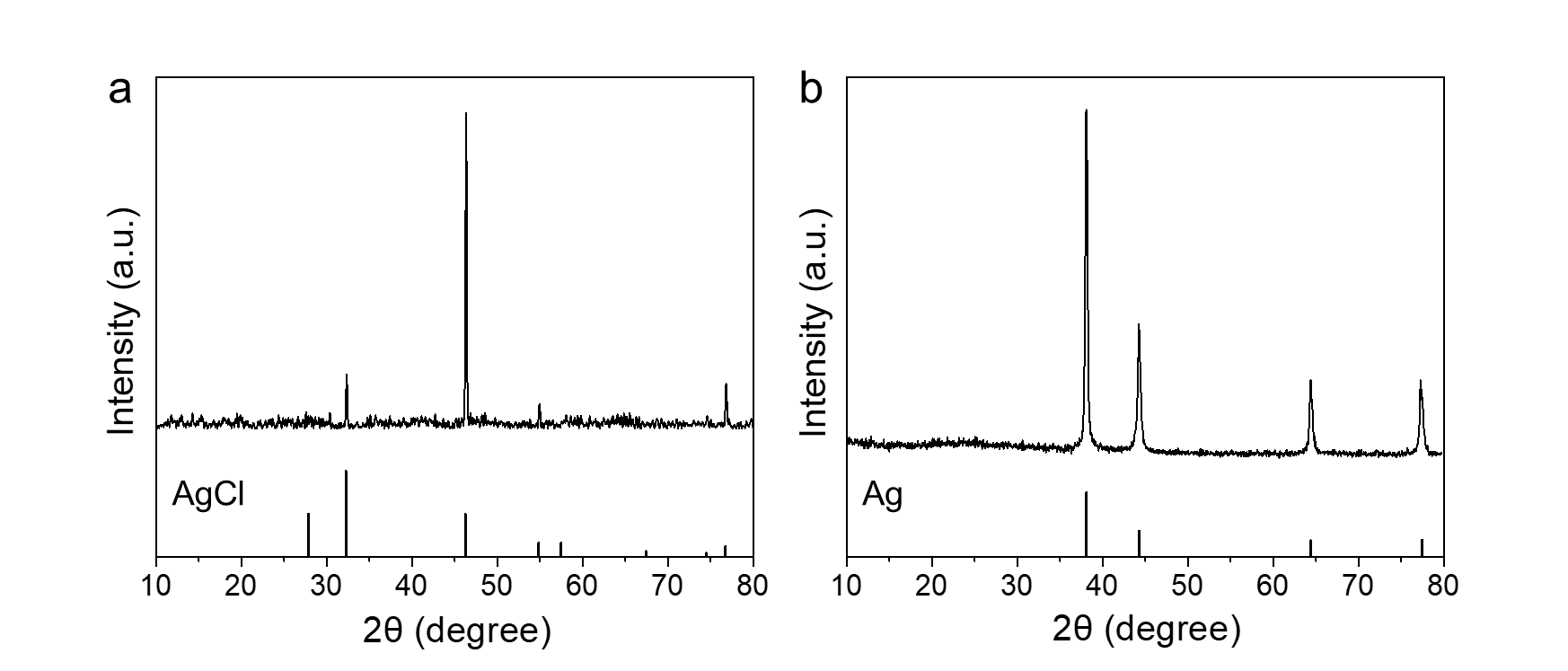


Figure S8. X-ray Diffraction results of the precursor AgCl (a) and NP Ag after RID (b).
